# An iterative approach to developing a multifaceted implementation strategy for a complex eHealth intervention within clinical practice

**DOI:** 10.1186/s12913-023-10439-1

**Published:** 2023-12-21

**Authors:** Renée V.H. IJzerman, Rosalie van der Vaart, Linda D. Breeman, Karin Arkenbout, Mike Keesman, Roderik A. Kraaijenhagen, Andrea W.M. Evers, Wilma J.M. Scholte op Reimer, Veronica R. Janssen

**Affiliations:** 1 Health, Medical and Neuropsychology Unit, Faculty of Social and Behavioural Sciences, Leiden University, Wassenaarseweg 52, Leiden, 2333 the Netherlands; 2 Centre of expertise Health Innovation, The Hague University of Applied Sciences, Den Haag, the Netherlands; 3 Department of Cardiology, Tergooi MC, Blaricum and Hilversum, the Netherlands; 4 Vital10, Amsterdam, the Netherlands; 5 Department of Cardiology, Amsterdam UMC, University of Amsterdam, Amsterdam, the Netherlands; 6 Department of Cardiology, Leiden University Medical Centre, Leiden, the Netherlands

**Keywords:** Implementation, Implementation strategy, Routine practice, eHealth, eHealth intervention, Delivery of health care

## Abstract

**Background:**

The number of complex eHealth interventions has increased considerably. Despite available implementation theory outlining well-designed strategies, implementing complex interventions within practice proves challenging and often does not lead to sustainable use. To improve sustainability, theory and practice should be addressed during the development of an implementation strategy. By subsequently transparently reporting the executed theory-based steps and their corresponding practice findings, others can learn from these valuable lessons learned. This study outlines the iterative approach by which a multifaceted implementation strategy for a complex eHealth intervention in clinical practice was developed, tested and refined.

**Methods:**

We implemented the BENEFIT program, an advanced eHealth platform with Personal Health Portal facilitating healthy living in cardiac patients. In six iterative phases alternating between theory and practice, the implementation strategy was developed, tested and refined. The initial implementation strategy (phase 1) was drawn up using the Implementation model and RE-AIM. Subsequently, this strategy was further updated in brainstorming sessions and group discussions with twenty key stakeholders from three cardiac care centres and then evaluated in a pilot (phases 2 and 3).

**Results:**

The pilot of the program led to the identification of (context-specific) key challenges in practice (phase 4), which were subsequently connected back to broader theory (phase 5) using the Consolidated Framework of Implementation Research (CFIR). In the final phase, practice recommendations tackling the key challenges were formulated (phase 6) based on CFIR theory, the CFIR-ERIC Matching Tool, and stakeholders’ input and feedback. These recommendations were then added to the refined strategy. Thus, executing this approach led to the realisation and use of a multifaceted theory-informed practice-based implementation strategy.

**Conclusion:**

This case study gives an in-depth description of an iterative approach to developing an evidence-based, practice-tailored strategy for implementing a complex eHealth intervention in cardiac care. As such, this study may serve as a blueprint for other researchers aspiring to implement complex eHealth interventions within clinical practice sustainably.

**Supplementary Information:**

The online version contains supplementary material available at 10.1186/s12913-023-10439-1.

## Background

Reducing cardiovascular disease (CVD) is a global health priority. Through innovative use of technology-based interventions, known as complex eHealth interventions, patients with CVD can be supported in improving their health and lifestyle behaviour at various stages. During hospitalisation (phase I), eHealth can offer tailored information and resources, preparing patients for future trajectories and providing personalised health information and communication channels with healthcare providers. During cardiac rehabilitation (CR) (phase II), eHealth interventions and remote monitoring can stimulate recovery. They significantly benefit duration of physical activity, daily steps, quality of life, and re-hospitalisation during this phase [1]. As achievements of CR should be consolidated in the long term (phase III), eHealth interventions offer ongoing support, medication reminders, and telehealth consultations to ensure continuity of care to further empower patients and improve CVD healthcare delivery. During this phase, research has shown significant benefits of eHealth interventions on health outcomes; they stimulate physical activity, improve exercise, increase quality of life, and decrease systolic blood pressure [2].

In the last decade, the number of eHealth interventions has increased considerably. Unfortunately, these initiatives often do not lead to sustainable use, as implementation within practice proves challenging, even when using available implementation theory. Lack of knowledge about conditions for sustainable use within practice has been labelled as “one of the most significant translational research problems of our time” (p.2) [3]. Understandably, implementing innovative and complex interventions has been shown to pose several challenges. One primary reason is that deployment of complex eHealth interventions is characterised by using multiple interacting features or user groups, within the context of social systems and structures that evolve continuously [3, 4]. These external factors significantly influence individual characteristics and behaviours [4]. Furthermore, the complexity arises from the multiple interplay of behavioural, technological, and organisational components [5], which have unclear, non-linear dynamics and thus require coordination on many levels to become effective [5–7]. Other challenges regularly include costs, lack of fit within the organisation, stakeholders not collaborating closely, and poor planning [6, 8–11].

Research has shown that multifaceted implementation strategies tailored to the targeted environment and population contribute to the adoption of interventions and sustainable use in the long term [12–14]. An implementation strategy has been described as “a systematic intervention process to adopt and integrate evidence-based health innovations into usual care.” (Powell et al., 2012, p.124) [15]. Implementing interventions using well-designed multifaceted implementation strategies is especially relevant for complex eHealth interventions. Moreover, as recently stated by the Standards for Reporting Implementation Studies (StaRI) initiative [16, 17], there is an urgent need for transparent and thorough reporting of strategies used to implement complex health interventions. Since then, a growing group of researchers and stakeholders reported the development and execution of their implementation strategies to let others build on their lessons learned [18, 19]. This way, the adoption and implementation of new interventions can become more effective and efficient, which contributes to improving health at the individual and societal levels [20]. Building on the call for transparency and thorough reporting, our case study describes the iterative approach by which a multifaceted implementation strategy for a complex eHealth intervention in routine cardiac care was developed, tested and refined.

### The BENEFIT program

The BENEFIT for all intervention program (in short: BENEFIT program) is a complex eHealth intervention, designed as a blended care approach, aimed at promoting and supporting sustainable healthy living among people with CVD in the Netherlands. Patients are actively engaged in following online lifestyle interventions and challenges. In addition, healthcare providers play a pivotal role by providing healthcare services, guidance, and coaching throughout the entire process.

Core to the BENEFIT program is access to an advanced eHealth platform with Personal Health Portal (PHP). The PHP provides access to (evidence-based) lifestyle interventions, daily goal monitoring, personal coaching, and a rewards program aimed at encouraging a wide range of health behaviours and adherence. These lifestyle interventions can be tailored to end-users’ needs and preferences. Moreover, self-management of a healthy lifestyle is promoted by encouraging patients to measure health indicators (such as blood pressure) themselves at home [21, 22]. Patients enrolled in cardiac rehabilitation (CR) receive the BENEFIT program from the start of their CR trajectory (phase II) as an addition to standard care. This way, healthcare providers can provide patients with information, feedback, and remote healthcare, and facilitate them in selecting suitable lifestyle interventions. After the standard rehabilitation program has ended, patients can continue to use the BENEFIT program (phase III). As a structured process combines patient input and clinical assessment to align lifestyle interventions with the patient’s needs, this process can therefore take place without the guidance of a healthcare provider. Patients are asked to complete questionnaires that focus on health-related topics and actively monitor their health-related values from the start and throughout the use of the program. These data serve as valuable data points for personalisation. For instance, if a patient indicates smoking in their questionnaire responses, this information is captured and flagged within the system, and the patient is then offered a smoking cessation program as part of their personalised intervention plan.

### Selection of suitable theoretical approaches

**Fig. 1:**
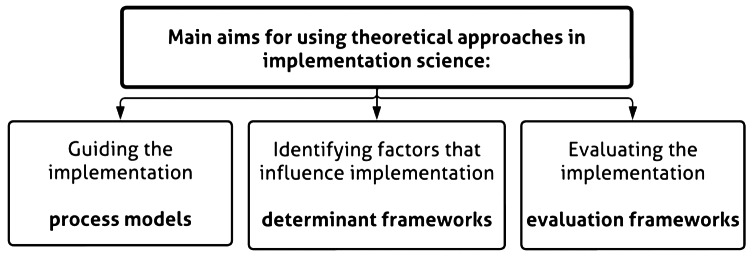
Main aims for using theoretical approaches in implementation science, described by Nilsen et al. (2015)

As the BENEFIT program concerns a complex eHealth intervention implemented within routine cardiac care, it was crucial to develop a multifaceted implementation strategy firmly rooted in theory. Subsequently, the strategy needed to be adaptable to the unique dynamics of stakeholders’ environments, work settings, daily routines, and expectations. To establish a strong theoretical foundation, our selection of relevant implementation frameworks was guided by a review by Nilsen and colleagues [12] which identifies three aims of using theoretical approaches in implementation science (see Fig. 1). These goals are to guide the implementation using a process model, identify factors influencing the implementation process using a determinant framework, and evaluate the implementation using an evaluation framework. By choosing a multi-deployable approach for the development of the BENEFIT implementation strategy, support and guidance would be given during all parts of the process; the development and operationalisation of the implementation strategy, implementation of the intervention program in routine cardiac care, as well as adaptation of the developed implementation strategy.

Three frameworks were selected as the foundation for the BENEFIT implementation strategy: the Implementation model [23] (process model), the Consolidated Framework of Implementation Research (CFIR) [24] (determinant framework), and RE-AIM [25, 26] (evaluation framework). The Implementation model was selected to provide a systematic approach to implementation planning and execution. As leading authors within implementation science, Grol and Wensing’s Implementation model contains seven steps that guide the implementation of complex interventions within the healthcare setting while considering the practice’s complexity; (1) Development of proposal for change, (2) Analysis of actual performance, targets for change, (3) Problem analysis of target group and setting, (4) Development and selection of strategies and measures to change practice, (5) Development, testing and execution of implementation plan, (6) Integration of changes in routine care, and (7) (Continuous) evaluation and (where necessary) adapting plan. As combining top-down and bottom-up approaches is essential to realise implementation within practice, all steps of the Implementation model were considered during the development and refinement of the BENEFIT implementation strategy.

Subsequently, the CFIR was selected to identify key factors and conditions influencing implementation. The CFIR is a meta-theoretical framework; it comprises several determinant frameworks and relevant theories from different disciplines, leading to the identification of five domains and 39 associated constructs, namely (1) Intervention Characteristics: such as Complexity and Adaptability, (2) Outer Setting: such as Peer pressure and External policies and incentives, (3) Inner Setting: such as Network and communications and Culture, (4) Characteristics of Individuals: such as Knowledge and beliefs about the intervention and Self-efficacy, and (5) Process: such as Planning and Engaging. As the CFIR facilitates systematic analysis and consistent use of its constructs, it can be adapted to many contexts and has often been used to implement and evaluate complex health implementations. Therefore, the CFIR is suitable for identifying influencing factors during the implementation process of the BENEFIT program. Subsequently, the selection of discrete implementation strategies (i.e., frequently described implementation actions consisting of one specific process or action to facilitate implementation, such as technical assistance, digital reminders, and educational training) is possible. By adding discrete implementation strategies to the implementation strategy, influencing factors within practice can be anticipated, and further optimalisation of the multifaceted implementation strategy is realised. Finally, the widely used evaluation framework RE-AIM – an acronym for Reach, Effectiveness, Adoption, Implementation, and Maintenance – was selected to provide a systematic approach to the operationalisation of different aspects of the implementation evaluation by using their online planning tool [27]. To illustrate, indicators for Implementation included a timeline for implementation of the program, user (patient) inclusion, interim feedback moments and evaluations from both health care provider perspective and patient perspective, and the number of adjustments that are made.

### Aim of the study

This case study describes the iterative development, evaluation, and improvement of the implementation strategy for the BENEFIT program, a complex eHealth intervention within routine cardiac care. The objectives of our case study are:

1. Combining theoretical approaches of implementation science within practice, and alternating between theory and practice during the development process of the implementation strategy, to identify key challenges – named key factors – negatively influencing the implementation within practice, and anticipate them by adding evidence-based discrete implementation strategies.
2. Using this iterative approach, the translation of a theory-based strategy into a strategy that also works in practice because it fits the stakeholders’ environment, working conditions, daily operations, and expectations can be realised.
3. Transparently and thoroughly reporting of the entire development process of the BENEFIT implementation strategy, our presented approach can be used as a blueprint for other researchers who aspire to implement a complex eHealth intervention sustainably.

## Methods

### Developmental phases of the BENEFIT implementation strategy

**Fig. 2:**
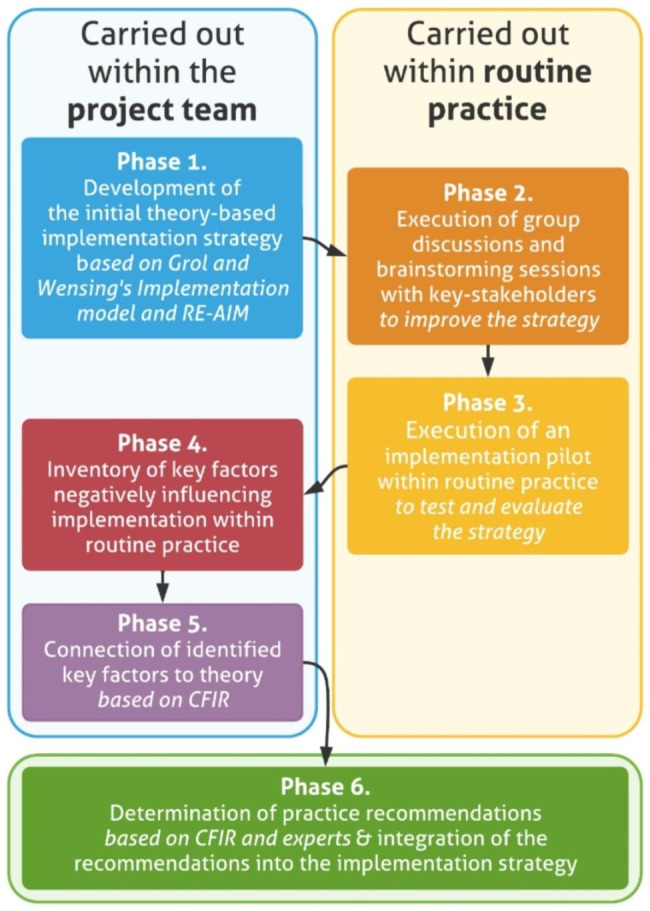
Six-phase approach to developing the BENEFIT implementation strategy

The development of the implementation strategy comprised six chronologically executed phases in which the three selected implementation frameworks are integrated. Figure 2 shows all development phases of the implementation strategy. The phases in which elaboration or application of theory takes place, namely 1, 4, 5, and 6, are solely carried out by members of the BENEFIT research project team (due to a public-private partnership, the project team consists of both researchers and health care providers). The remaining phases, namely 2 and 3, are carried out in collaboration within organisations within routine practice, namely two CR clinics and a hospital’s cardiac department, to improve, test, and evaluate the implementation strategy.

### Phase 1. Development of the initial theory-based implementation strategy

**Fig. 3:**
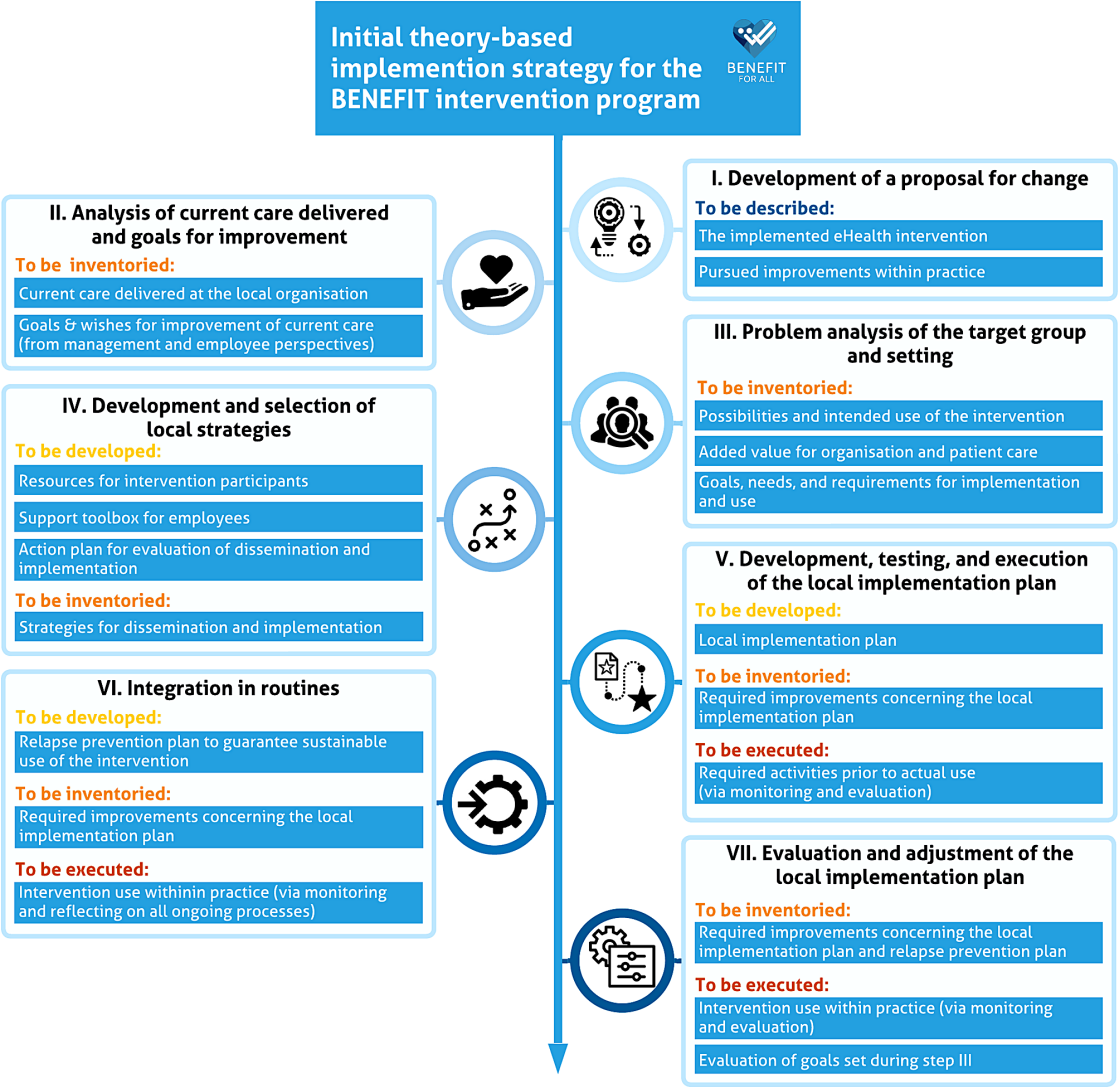
Result of phase 1: the initial theory-based implementation strategy

The foundation of the initial implementation strategy, carried out by three researchers from the BENEFIT research project team in 2018, was based on combining the Implementation model by Grol and Wensing and the evaluation framework RE-AIM. All seven steps of the Implementation model were elaborated to develop a clear step-by-step plan for the initial implementation strategy. In addition, RE-AIM was used by integrating its relevant implementation outcome indicators into the strategy [27] and expanding and substantiating the parts in the 7-step process that focus on adoption, implementation, and maintenance. Figure 3 depicts the initial implementation strategy (see Phase 1 in Fig. 2).

### Phase 2. Execution of group discussions and brainstorming sessions *to improve the strategy*

After developing the initial strategy, input from twenty key stakeholders was asked to further operationalise and adapt the implementation strategy (see Phase 2 in Fig. 2). To illustrate, in our case study, three organisations were involved during this phase (spread over a year from the second half of 2018): two CR clinics and a hospital’s cardiac department. These organisations met the established inclusion criterion, namely financial agreement by the organisation’s management regarding purchasing the necessary software for the BENEFIT program. Key stakeholders held various positions, namely, (i) health care professional roles (e.g., cardiologists, internists, (specialised) nurses, surgeons, a medical doctor, a physician assistant, a lifestyle coach, and physiotherapists), (ii) administrative and supportive roles (such as clinic assistants), and finally (iii) the combination of management positions with health care positions (e.g., general directors, directors of business development and operations, combined with the position of, for instance, cardiologist, internist, physiotherapist, and nurse). Depending on their position and the specific focus of the sessions, all key stakeholders participated in (part of) six group discussions and brainstorming sessions to provide input and feedback. During these sessions, members of the BENEFIT research project team executed and tested steps I to IV of the initial implementation strategy. These sessions aimed to assess the overall practicality of the developed initial implementation strategy by the BENEFIT research project team members involved, and to directly discuss perceived barriers or unclarities in the operationalisation of the developed strategy with the participating stakeholders. Among others, it was found that healthcare providers desired more comprehensive information regarding the BENEFIT program. They also wished for clear communication that lifestyle change is not a replacement for current care, but rather a complement that can lead to even better outcomes. Additionally, they sought an extensive explanation of how behavioural and lifestyle changes operate at the meta-level. By executing Phase 2 of the six-phase approach, the first round of improvement of the operationalisation of the initial strategy, tailored to the needs and contexts of the stakeholders within practice, was realised. These adjustments and improvements made by the BENEFIT research project team members were set out in different working documents.

### Phase 3. Execution of an implementation pilot *to test and evaluate the strategy*

After improving the initial implementation strategy, an implementation pilot took place at one of the CR clinics in 2019 (see Phase 3 in Fig. 2). Eleven employees were involved, namely health care providers and support, administrative and management employees. To demonstrate, the implementation pilot of our case study started with an introductory meeting in which BENEFIT research project team members presented the BENEFIT program presentation that was specifically developed for future introductory meetings with other organisations. At the beginning of the introductory meeting, employees were asked whether they agreed to listen to the presentation alternately from two perspectives. In the largest part of the meeting, they were asked to listen to the presentation and to learn about the BENEFIT program and its added value for their organisation. However, the presentation would be paused a few times to ask employees to put on their cardboard party hats renamed ‘thinking hats’ to reflect on how they liked a specific part of the presentation and where improvement was desired. All employees provided input and feedback on the process and content of the introductory meeting.

Following the introductory meeting of the implementation pilot, those employees responsible for the local implementation process carried out the first steps of the initial implementation strategy within practice. To illustrate, local employees formed several small working groups to develop, pilot test, and evaluate specific materials (e.g., patient and health care provider material and resources such as supporting documents, developed protocols, and manuals). During several working group sessions, which were spread over several months following the introductory meeting, members of the BENEFIT research project team were present to report employees’ responses directly to help improve the implementation strategy, and by scheduling a fixed feedback moment at the end of each working group session. For example, one of the sessions focussed on evaluating patients’ referral to the BENEFIT program which had taken place in the weeks before. Patients were referred to the BENEFIT program with a preconceived advice communicated by health care providers, after which they handed over a registration voucher and information folder to the patient. Afterwards, these patients were asked to provide feedback on both the process and the materials. By evaluating the experiences from patients’ and health care providers’ perspectives, it was possible to optimise the developed materials further. Hence, by conducting Phase 3, it was possible to further test and evaluate the developed implementation strategy in collaboration with key stakeholders within practice.

## Results

### Phase 4. Inventory of key factors negatively influencing implementation within routine practice

To further tailor the initial implementation strategy to practice, five members of the BENEFIT research project team made an overview of all influencing factors in practice observed during Phases 2 and 3 (which was set out in a working document shared internally). These influencing factors comprised positive ones that facilitated implementation success and thus did not need resolving, and negative ones that needed to be changed to increase implementation success. In several meetings with members of the BENEFIT research project team and other stakeholders, the barriers were discussed and analysed according to their perceived negative influence on implementation success. Thus, four essential barriers, names key factors, were identified as leading influencers that needed to be anticipated during the implementation. To illustrate, one of these key challenges constituted the finding that implementing all features of the BENEFIT program proved a challenge in some organisations, as some elements were better suited to certain settings than others. Table 1 describes the key factors and their influence during the implementation within routine cardiac care (see Phase 4 in Fig. 2).

**Table 1:** Result of phase 4: key factors negatively influencing implementation of the BENEFIT program

Identified key factors	Explanation of negative influence during the implementation
*Implementing all features of the BENEFIT program proved undesirable within some organisations.*	• While pilot testing the program, some elements of the program appeared better suited to certain settings than others. • Some organisations preferred blended care – a combination of health care on location and program use – which required certain program adaptations.
*The way the initial implementation strategy was presented to the target audience proved unsuitable.*	• During the first stakeholders’ sessions, the presented process steps overwhelmed employees. • They became demotivated or annoyed and stated they did not have enough time available for the required preparation time before using the intervention within practice.
*Unclear assignment of roles, responsibilities, and agreements * *proved an obstacle.*	• At the start of the implementation within some organisations, it was difficult to set clear goals and come to mutual agreements. • This brought insufficient clarity of the urgency and execution of specific tasks, which took speed out of the process and caused a decrease in motivation and commitment within the local team.
*Employees experienced that * *their management imposed the BENEFIT program on them.*	• Within some organisations, management had not first discussed the intervention’s relevance for employees or their patients, and had not involved their employees in deciding whether to implement the program. • This made some employees feel unmotivated, not heard, seen, or taken seriously.

### Phase 5. Connection of key factors to theory

To further improve the implementation strategy and its effectiveness within practice, the identified key challenges, named key factors, were connected to relevant theory to guarantee scientific substantiation for subsequent strategy adjustments based on these key factors. In our case study, relevant constructs of the Consolidated Framework of Implementation Research (CFIR) that address the key factors were identified using the constructs’ detailed description and theoretically substantiated rationale for inclusion in CFIR. To reach a consensus on all relevant constructs, three researchers of the BENEFIT research project group discussed the rationale for possible influencing constructs for each key factor. Table 2 describes all key factors and their identified relevant domains and constructs (see Phase 5 in Fig. 2). For example, the first identified key factor, “Implementing all features of the BENEFIT program proved undesirable within some organisations.”, was connected with, among others, construct D. Adaptability (of domain 1. Intervention Characteristics). Since some organisations preferred blended care, program adaptations such as the integration of the local technological patient system with the patient system of the BENEFIT program were required. This led to the conclusion that the adaptability of an intervention program is an important point of attention, since failure to address context-specific needs during implementation reduces the likelihood of successful program implementation.

**Table 2:** Result of phase 5: Key factors connected to relevant CFIR domains and constructs

Key factors within practice	CFIR domains	CFIR constructs
*Implementing all features of the BENEFIT program proved undesirable within some organisations.*	**Intervention characteristics**	D. Adaptability E. Trialability F. Complexity
	**Characteristics ** **of individuals**	B. Self-efficacy C. Individual stages of change
	**Process**	A. Planning C. Executing
*The way the initial implementation strategy was presented to the target audience proved unsuitable.*	**Intervention characteristics**	E. Trialability G. Design quality and packaging
	**Characteristics of individuals**	B. Self-efficacy E. Other personal attributes (motivation, learning style)
	**Process**	A. Planning C. Executing
*Unclear assignment of roles, responsibilities, and agreements * *proved an obstacle.*	**Inner setting**	D. Implementation climate
	**Characteristics of individuals**	B. Self-efficacy E. Other personal attributes (motivation)
	**Process**	A. Planning B. Engaging
*Employees experienced that their management imposed the BENEFIT program on them.*	**Intervention characteristics**	A. Intervention source
	**Inner setting**	B. Networks and communications C. Culture D. Implementation climate
	**Characteristics of individuals**	A. Knowledge and beliefs about the intervention D. Individual identification with the organisation E. Other personal attributes (motivation)
	**Process**	A. Planning B. Engaging D. Reflecting and evaluating

### Phase 6. Determination of practice recommendations to optimise the implementation strategy

After connecting the identified key factors within practice to the corresponding CFIR domains and constructs described in Table 2, we identify additional discrete implementation strategies. These identified strategies, referred to as ‘practice recommendations’, were meticulously crafted to enhance and fine-tune the initial implementation strategy. This refinement was undertaken to ensure that the strategy was better aligned with and more anticipatory of the identified constructs. This way, further tailoring the initial strategy to the stakeholders’ local contexts was realised.

**Fig. 4:**
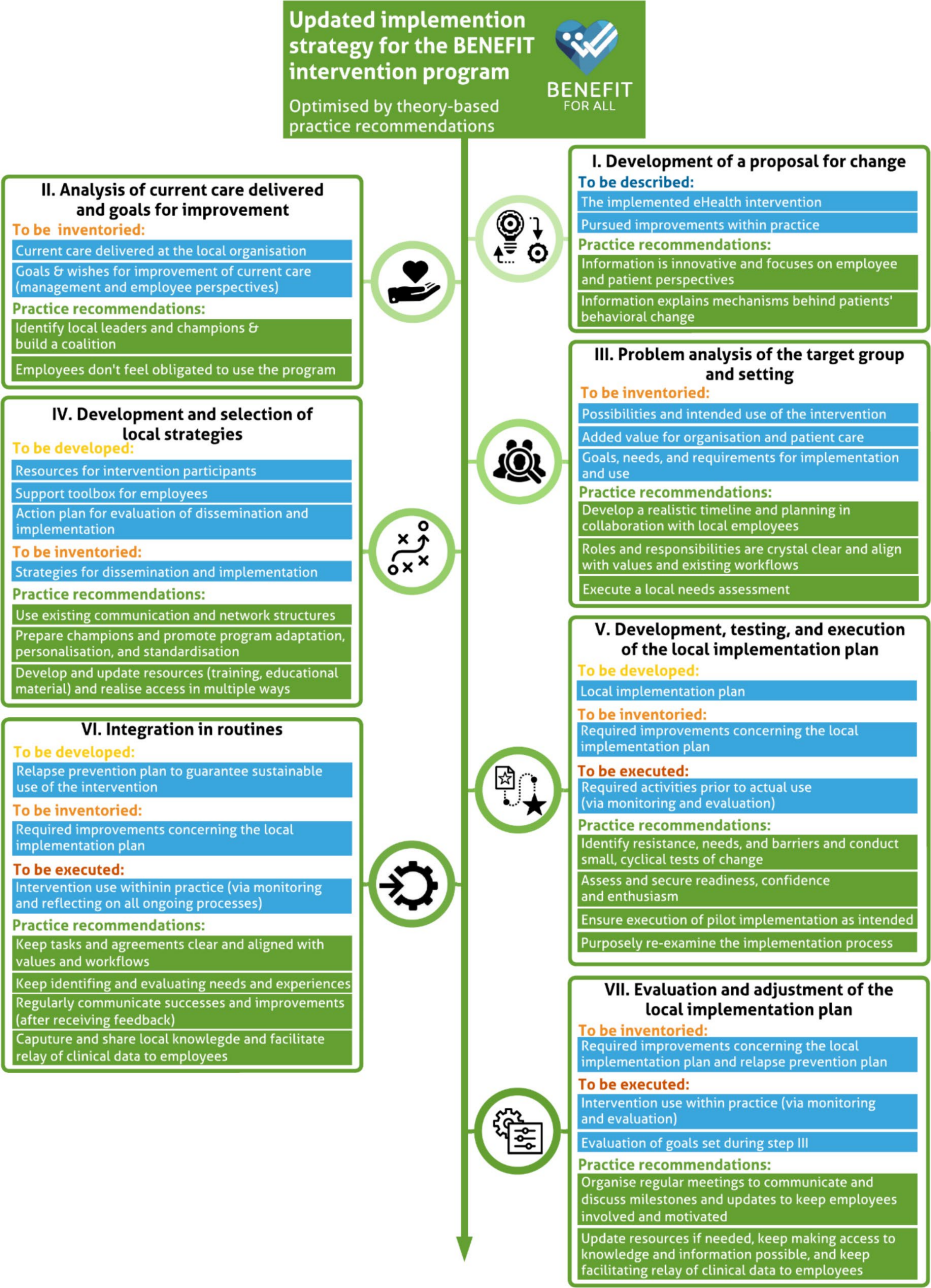
Final result of phase 6: the updated implementation strategy, optimised by theory-based practice recommendations

The practice recommendations were collected from both theory and practice by (i) searching the detailed description of all respective constructs (as described in Phase 5) on the official CFIR website for recommended evidence-based implementation strategies to anticipate these constructs, (ii) selecting evidence-based implementation strategies by using the CFIR-Expert Recommendations for Implementing Change (CFIR-ERIC) Matching tool (http://www.cfirguide.org/; accessed on September 6, 2021) [28, 29], and (iii) adopting already received input and feedback from key stakeholders during Phases 2 and 3. Subsequently, all practice recommendations were combined to identify which were suitable for multiple constructs. Three BENEFIT research project team members then discussed the practice recommendations and the best place to add them within the initial BENEFIT implementation strategy. Hence, by conducting Phase 6 of the six-phase approach, it was possible to select practice recommendations and integrate them into the initial implementation strategy, to better anticipate the key factors faced during the implementation. Table 3 describes all selected practice recommendations to anticipate the key factors. Figure 4 illustrates how these theory-based practice recommendations were integrated into the original initial implementation strategy, thus providing a new, updated implementation strategy (see Phase 6 in Fig. 2). To demonstrate, in our case-study, during the execution of Phase 2, healthcare providers expressed the need for a comprehensive understanding of how behavioural and lifestyle changes operate at a higher level. In response, we have incorporated the practice recommendation of “Information explains mechanisms behind patients’ behavioural change” in Step 1, which involves ‘Development of a proposal for change’. Additionally, in Step 4, which pertains to ‘Development and selection of local strategies’, we have added the practice recommendation of “Prepare champions and promote program adaptation, personalisation, and standardisation”. This practice recommendation aligns with the identified key factor “*Implementing all features of the BENEFIT program proved undesirable within some organisations*”. For a detailed description of all the steps of the updated implementation strategy, see Appendix A.

**Table 3:** Result of phase 6: practice recommendations to anticipate key factors

Key factors within practice	Practice recommendations to anticipate the identified key factors^1^
*Implementing all features of the BENEFIT program proved undesirable within some organisations.*	• Promote **adaptability of the intervention** (to anticipate the identified constructs 1D,1F,4C), **tailoring** of strategies (1D), conduct local needs **assessment** (1D,5A), **consensus** of **strategies** (1D) • Identify and prepare **champions** (4B,5A), assess for **readiness** and identify barriers and facilitators (1D,1E,5A,5C), conduct small **cyclical tests** of change (1E,1F) • Develop and implement tools for quality **monitoring** (5A,5C), develop a formal implementation **blueprint** (1F,5A) and a stage implementation **scale-up** (1F) • Develop a **dynamic training** (4B,4C), conduct **ongoing training** (1F,4B,5C), and provide ongoing **consultation** (4B)
*The way the initial implementation strategy was presented to the target audience proved unsuitable.*	• Adaptability (1G), assessment (5A), readiness (1E,5A,5C), blueprint (5A), cyclical tests (1E), dynamic training (4B), ongoing training (4B,5C), champions (4B,5A), consultation (4B), monitoring (1G,5A,5C) • Create learning **collaborative** (4B), develop educational **materials** (1G), model and **simulate** change (1E), provide technical **assistance** (5C), consumers **feedback** (1G), **re-examine implementation** (5C)
*Unclear assignment of roles, responsibilities, and agreements proved an obstacle.*	• Collaborative (4B), readiness (3D,5A,5B), assessment (3D,5A), blueprint (3D,5A,5B), ongoing training (4B), consultation (4B), dynamic training (4B), champions (4B,5A,5B), monitoring (5A) • Obtain formal **commitments** (5B), conduct local consensus **discussions** (3D), organise clinician implementation **team meetings** (3D), facilitate relay of **clinical data** to providers (3D)
*Employees experienced that their management imposed the BENEFIT program on them.*	• Champions (1A,3C,3D,4A,4D,5A), collaborative (3B,3C), feedback (1A,4E,5D), assessment (3D,5A), materials (4A), adaptability (1A), tailoring (3C), readiness (3C,3D,4D,4E,5A), blueprint (5A), monitoring (5A,5D), re-examine implementation (5D), discussions (3D,4D), team meetings (3B,3D,5D), clinical data (5D) • Recruit, design and train for **leadership** (3C,3D), build a **coalition** (1A,3B,4D), inform local **opinion leaders** (1A), use **workgroups** (1A,4A), and **educational meetings** (1A,4A), capture and share local **knowledge** (4A)

^1^The first time a practice recommendation is described, it is described fully with the most crucial keyword(s) displayed in bold. If the same practice recommendation is mentioned again to anticipate another construct that influences another key factor, only that recommendation's boldly presented keyword(s) is/are mentioned. This truncation of the practice recommendations is made to keep the table content manageable.

## Discussion

This study presents a multi-phased approach for developing an evidence-based and practice-tailored multifaceted implementation strategy. To date, reporting on the development of an implementation strategy for complex eHealth interventions is scarce. By thoroughly describing and reporting the executed iterative developmental process, our presented approach may be used as a blueprint for those who aspire to implement complex eHealth interventions within clinical practice sustainably. During this iterative development process, we have generated knowledge on some of the essential barriers, named key factors, to implementing the BENEFIT program within routine practice. Four key factors were identified, namely, (i) “Implementing all features of the BENEFIT program proved undesirable within some organisations.”, as some elements of the program were better suited to specific settings than others, (ii) “The way the initial implementation strategy was presented to the target audience proved unsuitable.”, as the number of presented theory-based process steps overwhelmed employees from practice, (iii) “Unclear assignment of roles, responsibilities, and agreements proved an obstacle.”, as it brought insufficient clarity of the urgency and execution of specific tasks, which took speed out of the implementation process, and (iv) “Some employees experienced that their management imposed the BENEFIT program on them.”, as their management had not involved them in deciding whether to implement the program.

The process of developing and refining a sustainable multifaceted implementation strategy for the BENEFIT program proved to be quite time-consuming. This was due to several aspects, including selecting suitable implementation frameworks for the initial multifaceted implementation strategy, maintaining contact with the involved organisations within practice, managing and evaluating all implementation phases performed within practice, identifying and anticipating influencing key factors within practice, selecting suitable implementation strategies to anticipate these emerging key factors, and reaching consensus within the BENEFIT research project team throughout the entire development process. However, utilising this iterative approach was vital to realise a practical, realistic, and tailored strategy. It helped confirm which parts of the initial strategy were appropriate and suitable for the local implementation environment, and in addition helped to identify key factors that negatively influenced implementation within practice. Implementation of the BENEFIT program would have been less successful without anticipating these identified key factors using carefully selected additional evidence-based implementation strategies. Among others, studies have shown that risks of failed implementation are reduced by managing the implementation process adequately, adapting the intervention program if needed, aligning new roles and responsibilities to existing workflows, and ensuring optimal preparation before using the intervention program within daily practice [9]. To demonstrate, in our case study, the second key factor “The way the initial implementation strategy was presented to the target audience proved unsuitable.” was anticipated by developing a different practical strategy that fitted our stakeholders’ thinking and working method in collaboration with them. In terms of content, the strategy was still theory-based, however, the way in which the strategy was presented to local organisations was significantly different. To illustrate, after consultation with several key stakeholders, our initial seven-step implementation strategy was conveyed to the local organisations in a simplified three-phased format, namely the self-confidence phase (a), self-regulation phase (b), and self-management phase (c), as these phases are commonly used within patients’ CR trajectories and are thus recognisable for the employees from the intended local organisations. Importantly, the content of the strategy remained unchanged; only the mode of communication to employees in the workplace was adapted. This modification, however, rendered the presented theory-based initial implementation strategy more practice-tailored by being recognisable, understandable, and manageable for those involved. In the future, it should become apparent whether this indeed is an effective approach to use within practice.

### Lessons learned for future implementation processes within routine practice

The execution of our case study has taught us several lessons. When developing a multifaceted implementation strategy for a complex eHealth intervention, we experienced that it is essential to alternate between theory and practice to create a tailored, realistic, and feasible strategy. Using such an iterative approach ensures that the final multifaceted implementation strategy is theoretically substantiated and fits well with the local organisations where the program needs to be implemented sustainably. Even though using such an approach can be experienced as inefficient at the time, it is nevertheless strongly recommended by experts because tailoring increases the adherence of healthcare providers to an intervention by up to 14% [30]. Our presented approach, the six-phase development process, may be a blueprint for researchers who aspire to implement a complex eHealth intervention successfully and sustainably. It is worth noting that significant intervals naturally occurred in our approach’s development process, as each phase required thorough establishment, elaboration, practical implementation, and findings processing before determining the next phase(s). When utilising a well-developed approach like our six-phase development process, the phase completion timeline substantially shortens. For example, the six phases of our approach can be completed in 4 to 6 months, this includes part of implementation.

Furthermore, when developing an implementation strategy, we found that it is crucial to communicate all implementation steps in a recognisable and manageable way to the intended local organisations. Using this approach increased adherence to the outlined implementation strategy. Keeping the implementation steps recognisable and manageable can be realised by, for example, conveying it to local organisations in a simplified format that fits the thinking and working method of the stakeholders. This is possible by, for instance, matching the implementation process as closely as possible to a working method that is familiar to employees within the targeted field.

Concerning the execution of an implementation strategy, our research project team has learned several valuable lessons. When executing an implementation strategy, we learned the importance of paying significant attention to successfully communicating the program’s added value for all different groups involved (i.e., patients, healthcare providers, and other stakeholders) as straightforward as possible to the local organisations that will offer the program to patients. We experienced that if employees involved are not convinced of the program’s added value, their effort and investment needed to practise new tasks will be insufficient to optimise and consolidate the new workflow, regardless of the potential success of an intervention program. Therefore, we realised that it is essential that the program’s implementation process will be facilitated by involving stakeholders from the field as early as possible to consider their needs and wishes and anticipate potential challenges within organisations.

Finally, when pilot testing an intervention program within local organisations, we observed the value of ensuring proper embedding of new tasks into pre-existing workflows and secure employees’ readiness, confidence, and enthusiasm concerning the use of the program. The realisation of these factors significantly influenced whether the program’s roll-out will be successful and, therefore, also influenced the implementation and use of the program in the long run.

### Strengths and limitations of the study

One of the strengths of our case study was the theoretical foundation used to develop the BENEFIT implementation strategy. By applying three theoretical implementation frameworks, it was possible to create an implementation strategy that guided the implementation process, helped identify factors that influenced the process, and provided guidance on how to adapt the implementation strategy. Furthermore, by choosing to use an iterative approach for the development of the implementation strategy, it was possible to alternate between theoretical frameworks and clinical practice to tailor the theory-based implementation strategy to the stakeholders’ environment, working conditions, daily operations, and expectations.

A limitation of our study relates to generalisability, as certain findings from practice constituted in our case study do not necessarily apply to other implementation practices. To illustrate, this research project was performed and executed in Netherlands. It is therefore important to note that different countries and cultures may require unique approaches for eHealth development and implementation. Also, our identified key factors do not mention the importance of financial support since one inclusion criterion for participating organisations was financial agreement by the organisation’s management. Funding, however, was an important determinant of whether to implement the BENEFIT program within several other organisations not included in this case study. However, as our main aim was to transparently describe the iterative development process of the BENEFIT implementation strategy to serve as a blueprint for other researchers, the described findings of our specific case study were used for illustrative purposes, and are thus inherently not generalisable.

### Future research and implications for practice

Concerning future research and implications for practice, various follow-up steps may be desirable. To begin with, in collaboration with stakeholders, it may be valuable to focus on ways to present the developed six-phase approach to organisations, for example, by using a website or (digital) toolkit where organisations can consult and go through all steps of the implementation approach. This further development of the approach ensures that it is made accessible to others interested. By allowing more people to use an approach that has taken several years to develop and refine, other researchers and healthcare providers from within practice are no longer obligated to invest significant time thinking out a suitable iterative development process themselves.

On top of that, by gaining other user experiences, the content of the approach can be further optimised for practice. Thereby, follow-up research can also focus on the financial aspect of using the tool, specifically the required time investment and commitment of all those involved when using this approach. In doing so, it can be examined whether the benefits of using the approach, namely sustainable implementation of a healthcare innovation within practice, outweigh the financial investment.

Finally, in developing the BENEFIT implementation strategy, we initially focused on a theory-based approach, leveraging implementation science frameworks and models to guide our efforts. However, it is vital to acknowledge that implementing and deploying complex eHealth interventions involves various factors and user groups. As mentioned, complex eHealth interventions exist within a constantly changing social and structural context, significantly influencing individual behaviours and characteristics [3, 4]. Therefore, including a systems-thinking perspective is crucial to comprehend the complex interplay between an intervention and the environment in which it is implemented and evaluated. This broader perspective necessitates thoroughly examining factors such as acceptability, feasibility, scalability, transferability of the intervention across different contexts, and cost-effectiveness [7].

## Conclusions

This case study gives an in-depth description of an iterative approach to developing an evidence-based, practice-tailored strategy for the implementation of the BENEFIT program, a complex eHealth intervention in cardiac care. In six iterative phases moving from theory to practice and back, the implementation strategy was developed, tested, and refined. As such, this study may serve as a blueprint for other researchers aspiring to sustainably implement complex eHealth interventions within clinical practice.

### Electronic supplementary material

Below is the link to the electronic supplementary material.

Supplementary Material 1

## Data Availability

The data used and/or analysed during the current study are available from the corresponding author on reasonable request.

## Abbreviations

CVD: Cardiovascular disease
PHP: Personal Health Portal
CFIR: Consolidated Framework of Implementation Research
CFIR-ERIC: Consolidated Framework of Implementation Research-Expert Recommendations for Implementing Change
