# Supplementary material for: An iterative approach to developing a multifaceted implementation strategy for a complex eHealth intervention within clinical practice
Source: BMC Health Serv Res. 2023 Dec 21;23:1455. doi: 10.1186/s12913-023-10439-1 (PMC10740292; doi:10.1186/s12913-023-10439-1)
Supplement: Supplementary file 1 — Supplementary Material 1 [file 12913_2023_10439_MOESM1_ESM.docx]

**Appendix A. Detailed description of the updated implementation strategy (Phase 6)**

**Regarding step I. Development of a proposal for change**

As shown in Fig. 4, during step I of the implementation process, the project team is advised to describe a clear and comprehensive program outline of the implemented eHealth intervention, including a rationale for the program’s added value and its’ expected improvements for the targeted setting. Findings gathered during brainstorming sessions and the implementation pilot within practice led to identifying two additional discrete implementation strategies, described as practice recommendations. These strategies were then added to the updated implementation strategy. To illustrate, in our case study, it was found that it is preferable to provide program information from patient and health care provider perspectives to pique the audience’s interest in the program. This way, the program’s value to both groups is shown, encouraging health care providers to adopt the program. Moreover, it is recommended to provide more background information on the mechanisms behind lifestyle change (behavioural change) and elaborate on different layers (theory, practice, SES differences).

**Regarding step II. Analysis of current care delivered and goals for improvement**

When executing step II, two themes are advised to inventory at the local organisation: current patient care and goals and wishes for improving routine cardiac care. Throughout this inventory, perspectives of the management, health care providers, and other employees are covered. After execution of the inventory within practice, several additional practice recommendations were drawn up. We found, for example, that it is recommended to stimulate local leaders inside the organisation to take part in the process as drivers for change, keep local opinion leaders informed about plans for program implementation, and identify and notify suitable champions. Moreover, to increase effective implementation, we found that attracting employees to inspire, motivate, and enthuse others was effective, as was building a group to support one another when going through the process. Finally, it became apparent that creating an environment where employees feel free to use the program was very important, as imposing it from a management perspective appeared to hamper implementation success.

**Regarding step III. Problem analysis of the target group and setting**

Step III, as described in Fig. 4, comprises an inventory of several themes. First, employees are advised to discuss how the program will improve their patient care and workflows. Also, it is preferable to explore local possibilities further and subsequently identify concrete program implementation goals. Then, it is recommended to define all needs and requirements for successful implementation and use. Following the completion of the pilot, several practice recommendations were drafted. As an example, in our case study, it appeared that it is preferable to a) let all employees jointly prepare and approve a timeline and planning (taking a 5-10% delay into account) as well as role descriptions and resulting responsibilities and agreements that correspond to personal values and existing workflows, and b) identify and evaluate needs and associated challenges as this helps to anticipate them in the next step effectively.

**Regarding step IV. Development and selection of local strategies**

Step IV of the implementation process involves developing support material and compiling a list of local strategies for dissemination and implementation. First, a toolbox for employees (e.g., webinar, protocols for inclusion of participants, step-by-step plan with instructions for using the program) and materials for future participants (e.g., explanation of the program, instructions for registration) are developed. Furthermore, drafting an action plan for evaluating the dissemination and implementation process is recommended. Findings gathered during the pilot within practice led to the identification of several practice recommendations. By way of illustration, the following findings emerged from our evaluation: a) stimulate local implementation managers to state that the program can be customised, personalised, and standardised to meet employees’ needs, b) since employees differ in needs and requests for help, it is advisable to provide information in multiple ways and to update these resources regularly, c) by utilising existing communication and network infrastructures, chances of miscommunication and implementation delays will be reduced, and finally, d) by stimulating and preparing local champions to lead the implementation process and act as a source of information, employees are encouraged to engage in the process and receive support if necessary.

**Regarding step V. Development, testing, and execution of the local implementation plan**

As shown in Fig. 4, during step V, the local implementation plan is developed and tested to improve the developed implementation plan further. Several practice recommendations were added after piloting our initial implementation strategy. For example, we found that securing employees' readiness, confidence, and enthusiasm is important to roll out the intervention program successfully. Moreover, we experienced that it is advised to realise a pilot implementation that is executed as intended, and to regularly assess employees' resistance, needs, barriers, and enablers to re-examine and enhance the implementation process if necessary.

**Regarding step VI. Integration in routines**

At the start of step VI, the intervention program will be implemented within practice. As described, processes are actively monitored and reflected on to determine which ones need to be changed to optimise embedding within practice. Necessary improvements will be incorporated into the implementation plan. Over time, a relapse prevention plan will be developed with all involved to provide further support in pursuing sustainable implementation and usage of the intervention in practice. Based on practice and theory, practice recommendations were included. To demonstrate, in our case study, it was found that already drawn tasks and agreements should be revised if required to match the employee’s values and workflows. Also, needs, experiences, feedback, and successes are advised to be assessed regularly. In addition, it is recommended to routinely communicate gathered input, clinical data, or knowledge to keep employees informed and involved. By sharing improvements made in response to employees’ feedback, employees are assured that their suggestions are valued.

**Regarding step VII. Evaluation and adjustment of the implementation plan**

Finally, as shown in Fig. 4, the last step of the implementation strategy comprises further improvements to the implementation process, implementation plan, and relapse prevention plan. By monitoring and reflecting on all ongoing processes and evaluating the earlier specified goals, chances of long-term program integration into routine practice will be increased. Based on findings gathered from practice and theory, practice recommendations were added to the implementation strategy. By way of example, we found that it was strongly beneficial to a) keep resources regarding training and education updated regularly. Also, it is recommended to realise access to knowledge and information permanently and in various ways, and keep employees involved and motivated using frequent meetings. During these meetings, news, successes, and milestones can be communicated from an employee, organisation, and patient perspective, and discussing developments and conveying clinical data is recommended.
